# Ethanol Treatment Enhances Physiological and Biochemical Responses to Mitigate Saline Toxicity in Soybean

**DOI:** 10.3390/plants11030272

**Published:** 2022-01-20

**Authors:** Ashim Kumar Das, Touhidur Rahman Anik, Md. Mezanur Rahman, Sanjida Sultana Keya, Md. Robyul Islam, Md. Abiar Rahman, Sharmin Sultana, Protik Kumar Ghosh, Sabia Khan, Tofayel Ahamed, Totan Kumar Ghosh, Lam Son-Phan Tran, Mohammad Golam Mostofa

**Affiliations:** 1 Department of Agroforestry and Environment, Bangabandhu Sheikh Mujibur Rahman Agricultural University, Gazipur 1706, Bangladesh; ashimbsmrau@gmail.com (A.K.D.); abiar@bsmrau.edu.bd (M.A.R.); tofayela@gmail.com (T.A.); 2 Plant Pathology Division, Bangladesh Rice Research Institute, Gazipur 1701, Bangladesh; anikbge@gmail.com; 3 Department of Plant and Soil Science, Institute of Genomics for Crop Abiotic Stress Tolerance, Texas Tech University, Lubbock, TX 79409, USA; mdmerahm@ttu.edu (M.M.R.); skeya@ttu.edu (S.S.K.); son.tran@ttu.edu (L.S.-P.T.); 4 Institute of Biotechnology and Genetic Engineering (IBGE), Bangabandhu Sheikh Mujibur Rahman Agricultural University, Gazipur 1706, Bangladesh; irobyul@gmail.com (M.R.I.); sharminomi87@gmail.com (S.S.); 5 Faculty of Agriculture, Bangabandhu Sheikh Mujibur Rahman Agricultural University, Gazipur 1706, Bangladesh; protikbsmrau@gmail.com; 6 Department of Plant Pathology, Bangabandhu Sheikh Mujibur Rahman Agricultural University, Gazipur 1706, Bangladesh; sabia.khan60@gmail.com; 7 Department of Crop Botany, Bangabandhu Sheikh Mujibur Rahman Agricultural University, Gazipur 1706, Bangladesh; totan@bsmrau.edu.bd; 8 Department of Biochemistry and Molecular Biology, Bangabandhu Sheikh Mujibur Rahman Agricultural University, Gazipur 1706, Bangladesh

**Keywords:** antioxidants, ethanol, ionic balance, oxidative damage, photosynthesis, reactive oxygen species, salinity, soybean

## Abstract

Soil salinity, a major environmental concern, significantly reduces plant growth and production all around the world. Finding solutions to reduce the salinity impacts on plants is critical for global food security. In recent years, the priming of plants with organic chemicals has shown to be a viable approach for the alleviation of salinity effects in plants. The current study examined the effects of exogenous ethanol in triggering salinity acclimatization responses in soybean by investigating growth responses, and numerous physiological and biochemical features. Foliar ethanol application to saline water-treated soybean plants resulted in an enhancement of biomass, leaf area, photosynthetic pigment contents, net photosynthetic rate, shoot relative water content, water use efficiency, and K^+^ and Mg^2+^ contents, leading to improved growth performance under salinity. Salt stress significantly enhanced the contents of reactive oxygen species (ROS), malondialdehyde, and electrolyte leakage in the leaves, suggesting salt-induced oxidative stress and membrane damage in soybean plants. In contrast, ethanol treatment of salt-treated soybean plants boosted ROS-detoxification mechanisms by enhancing the activities of antioxidant enzymes, including peroxidase, ascorbate peroxidase, catalase, and glutathione *S*-transferase. Ethanol application also augmented the levels of proline and total free amino acids in salt-exposed plants, implying a role of ethanol in maintaining osmotic adjustment in response to salt stress. Notably, exogenous ethanol decreased Na^+^ uptake while increasing K^+^ and Mg^2+^ uptake and their partitioning to leaves and roots in salt-stressed plants. Overall, our findings reveal the protective roles of ethanol against salinity in soybean and suggest that the use of this cost-effective and easily accessible ethanol in salinity mitigation could be an effective approach to increase soybean production in salt-affected areas.

## 1. Introduction

Climate change-induced extreme environmental conditions, such as salinity, drought, temperature extremes, and waterlogging, pose serious challenges to global agriculture, threatening food security [1]. Furthermore, given the current water-scarce scenario, high saline water is increasingly being used to meet the growing need for water for agricultural production in many parts of the world [2,3]. Consequently, an increasing level of soil salinity has emerged as a paramount environmental problem, affecting around 3.6 billion hectares (Bha) out of 5.22 Bha of the world’s agricultural land, with an annual loss of USD 27.5 billion [2,4,5]. Plants grown in salt-contaminated soils have accumulated excessive toxic ions, resulting in a variety of morphological, physiological, and biochemical disturbances, such as ionic disparities, poor gas exchange performance, photosynthetic pigments loss, low water status, and excessive reactive oxygen species (ROS)-induced oxidative damage to cellular components [2,6,7,8,9]. Plants, on the other hand, employ a wide array of mechanisms to counteract the detrimental consequences of salt stress. For example, salt-exposed plants mount mechanisms to (i) reduce toxic ion accumulations in the aerial parts; (ii) limit the destruction of photosynthetic pigments; (iii) increase the accumulation of osmoprotectants, such as proline (Pro), free amino acids and soluble sugars; and (iv) enhance the activities of antioxidant enzymes, including superoxide dismutase (SOD), ascorbate peroxidase (APX), catalase (CAT), glutathione *S*-transferase (GST), and glutathione peroxidase (GPX), and the contents of nonenzymatic compounds, such as carotenoids, phenolic compounds, and flavonoids [2,7,8].

Soybean (*Glycine max*) is a nutritious, low-cost, and economically important crop belonging to the family Fabaceae, which contributes to nearly 70% of plant-based protein consumption and 29% of edible oil worldwide [10]. Furthermore, being a legume crop, the inclusion of soybean in crop rotation is an effective approach to replenishing soil fertility, strengthening soil-nutrient recycling, improving soil microbial activities, and increasing crop yield due to its biological nitrogen (N) fixation ability [11]. Due to its numerous benefits, soybean has become a globally coveted crop, and its demand is steadily increasing year to year [10]. In Bangladesh, soybean is cultivated on roughly 0.07734 million ha (Mha) of land out of 0.47874 Mha of the total oil-cropped areas [12], and soybean production is estimated to be around 0.14695 million tons (MT) every year [13]. The growing population, changing consumer eating habits, expansion of the bakery and food industries, and increasing need for soy meal for the livestock and fishing sectors are among the major factors driving up demand for soybean products in Bangladesh [14]. Bangladesh’s agricultural production systems were unable to produce enough soybeans, resulting in the import of 2.4 MT of soybeans in 2020, which is expected to rise to 2.65 MT in 2021–2022 (https://www.fas.usda.gov/data/bangladesh-oilseeds-and-products-annual-2) (accessed on 10 November 2021). In addition to limited arable land for soybean cultivation, soil salinity caused a significant decline in soybean production and seed quality in Bangladesh [15]. Elevated soil salinity perturbs the entire life cycle of soybean, from germination to plant growth, and the formation of nodule and seed yield [10], although the seedling stage of soybean is more vulnerable to salinity than the germination stage [16]. It is reported that salinity can impede soybean seed germination by 18% (at 2.09 dS m^−1^) to 70% (at >5 dS m^−1^) in salt-affected areas of Bangladesh [17].

To improve the salinity tolerance of soybean, various approaches, including gene discovery, breeding, and biotechnological strategies, are in practice [18]. Apart from the aforementioned strategies, developing simple and less expensive technologies for low-income nations such as Bangladesh, where insufficient funding in research and development discourages scientists from researching, and developing genetically modified crops, is still necessary. In this context, exploring the potential roles of external chemicals may offer an effective strategy in boosting plant resiliency against ever-changing environmental assaults. Ethanol has been demonstrated to improve chilling tolerance in rice (*Oryza sativa*) [19] and salt stress tolerance in rice and *Arabidopsis thaliana* [20]. The application of ethanol augmented the chlorophyll (Chl) content in *Arabidopsis* under salt stress while simultaneously lowering the buildup of ROS [20]. In line with this, exogenous ethanol supplementation has also been shown to upregulate the expressions of *APX1* and *APX2*, encoding APX, and ROS signaling-related transcription factor genes *ZAT10* and *ZAT12*, which were associated with salinity acclimatization responses in *Arabidopsis* [20]. Considering this clue, we also foresee that this affordable and easily accessible chemical ethanol could play an influential role in reducing salt-induced adverse effects on an economically important crop, soybean.

In this study, we examined whether ethanol could protect soybeans, as it did in other crops, from the adverse effects of salinity. If it does so, what are the underlying mechanisms that play vital roles in improving the salt tolerance potential of soybeans? Here, we examined the functions of ethanol in improving the salinity tolerance of soybean by evaluating the morphological, physiological, and biochemical features associated with (i) growth enhancement and biomass production, (ii) sodium ion (Na^+^) uptake and accumulation, (iii) photosynthetic pigment status, (iv) salt-induced oxidative stress, (v) antioxidant defense system, and (vi) osmotic adjustments.

## 2. Results

### 2.1. Ethanol Improves Phenotypic Appearance of Soybean Plants

**Figure 1:**
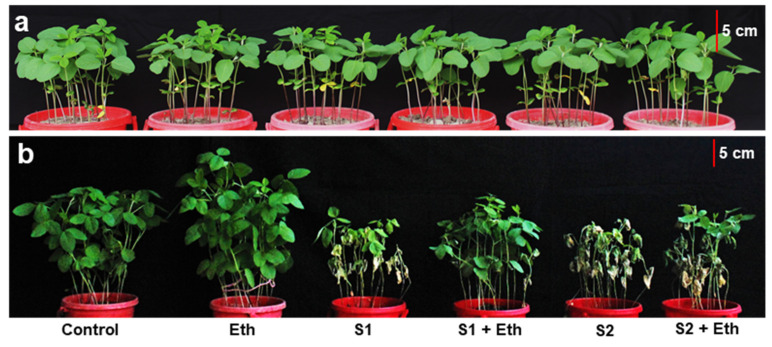

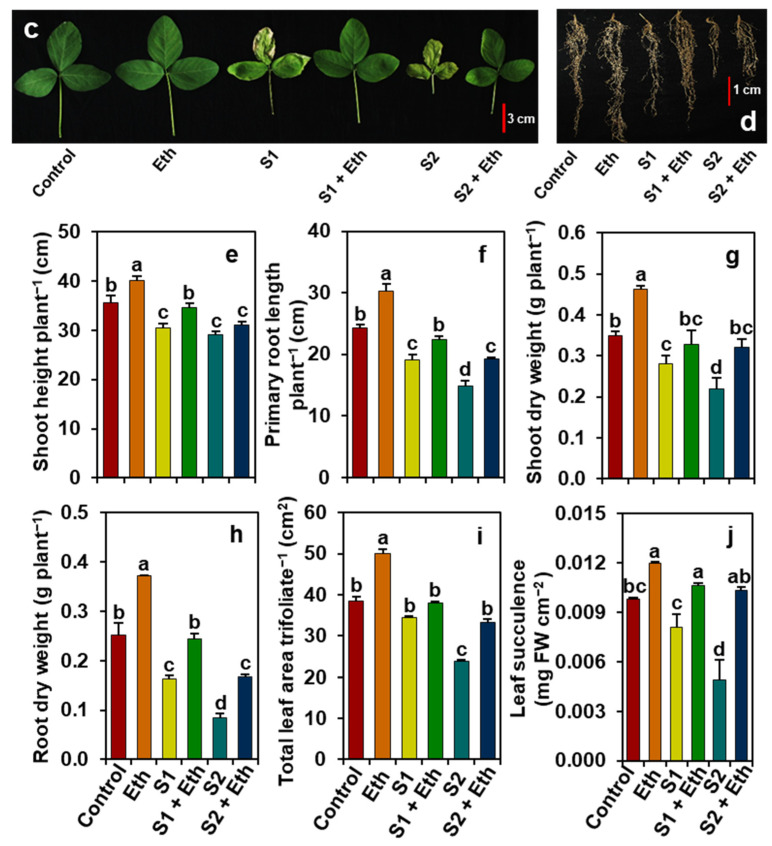
Effects of foliar-sprayed ethanol on growth-associated attributes of soybean plants under salt stress conditions. Representative photographs of soybean plants before stressed with salt (**a**) and after exposure to a gradient of salinity for 7 days (**b**). Close view of representative leaves (**c**) and roots (**d**) showing a positive effect of ethanol on salt-stressed soybean plants. (**e**) Shoot height, (**f**) primary root length, (**g**) shoot DW, (**h**) root DW, (**i**) total leaf area per trifoliate, and (**j**) leaf succulence of soybean plants exposed to different levels of salinity for 7 days in the presence and absence of ethanol. The statistically significant differences (*p* < 0.05) among various treatments are shown by different letters following the least significant difference (LSD) test. FW, fresh weight; DW, dry weight; Eth, 20 mM ethanol; S1, 8 dS m^−1^; S1 + Eth, 8 dS m^−^^1^ + 20 mM ethanol; S2, 16 dS m^−1^; S2 + Eth, 16 dS m^−^^1^ + 20 mM ethanol.

Soybean plants exposed to 8 dS m^−1^ (S1) and 16 dS m^−1^ (S2) salt stress for 7 days showed substantial phenotypic disruption, including stunted growth, wilting, early senescence, decolorization of leaves (turned to pale and yellow), and reduction in root length, as compared with the ‘Control’ plants (Figure 1a–d). By contrast, plants sprayed with 20 mM of ethanol (Eth) significantly reduced salinity-induced toxic effects in ‘S1 + Eth’ and ‘S2 + Eth’ plants, as manifested by their improved phenotypes, including less wilting and yellowing of leaves, delayed leaf senescence, and enhanced root length, when equated with the respective salt-stressed ‘S1’ and ‘S2’ plants (Figure 1a–d). Interestingly, under non-stressed conditions, the application of exogenous ethanol also improved the visual appearance of the shoots and roots in ‘Eth’ plants, in relation to the ‘Control’ plants (Figure 1a–d).

### 2.2. Ethanol Boosts Growth Attributes in Salt-Stressed Soybean Plants

‘S1’ and ‘S2’ plants, respectively, displayed noteworthy reductions in shoot height (by 14.41 and 18.34%), root length (21.37 and 39.04%), shoot dry weight (DW) (19.52 and 37.14%), root DW (35.10 and 66.23%), total leaf area per trifoliate (38.19% in ‘S2’), and leaf succulence (50.10% in ‘S2’), when compared with those of the ‘Control’ plants (Figure 1e–j respectively). Conversely, remarkable improvements in shoot height (by 13.66% in ‘S1 + Eth’), root length (16.72 and 29.89%), shoot DW (45.45% in ‘S2 + Eth’), root DW (48.98 and 96.08%), total leaf area per trifoliate (39.75% in ‘S2 + Eth’), and leaf succulence (32.05 and 110.68%) were manifested in ‘S1 + Eth’ and ‘S2 + Eth’ plants, respectively, when contrasted with the corresponding ‘S1’ and ‘S2’ plants (Figure 1e–j). Moreover, ‘Eth’ plants also showed enhancements in the height of shoot, length of root, root DW, shoot DW, total leaf area per trifoliate, and leaf succulence by 12.35, 24.25, 47.68, 32.38, 29.96, and 21.76%, respectively, compared with the corresponding data obtained from the ‘Control’ plants (Figure 1e–j).

### 2.3. Ethanol Protects Gas Exchange Features in Soybean Plants under Salt Stress

**Figure 2:**
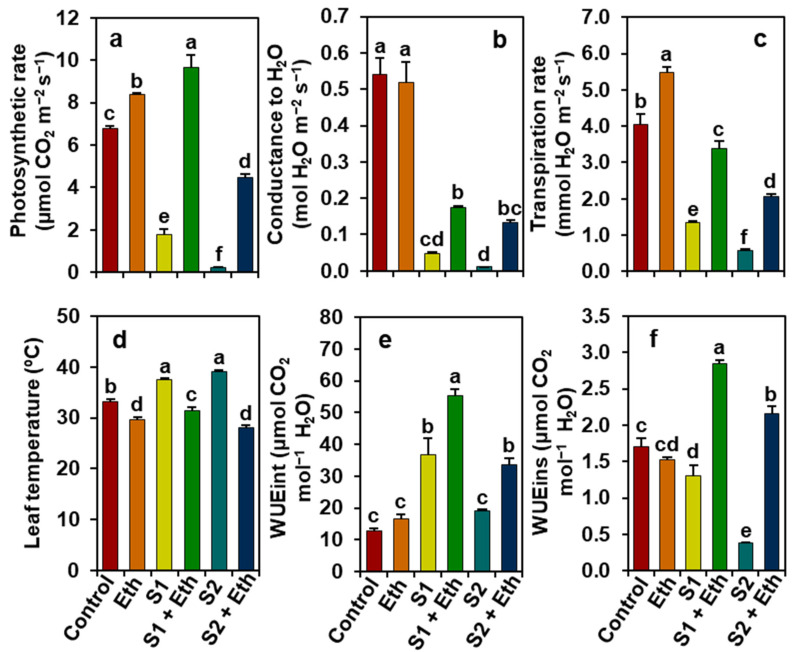
Effects of foliar-sprayed ethanol on (**a**) photosynthetic rate (*P_n_*), (**b**) stomatal conductance to H_2_O (*g_s_*), (**c**) transpiration rate (*E*), (**d**) leaf temperature (LT), (**e**) intrinsic water use efficiency (WUEint), and (**f**) instantaneous water use efficiency (WUEins) in the leaves of soybean plants exposed to a gradient of salinity for 7 days. The statistically significant differences (*p* < 0.05) among various treatments are shown by different letters following the least significant difference (LSD) test. Eth, 20 mM ethanol; S1, 8 dS m^−1^; S1 + Eth, 8 dS m^−1^ + 20 mM ethanol; S2, 16 dS m^−1^; S2 + Eth, 16 dS m^−1^ + 20 mM ethanol.

Relative to the ‘Control’ plants, ‘S1’ and ‘S2’ plants, respectively, showed remarkably decreased levels of photosynthetic rate (*P_n_*, by 74.03 and 96.81%), stomatal conductance to H_2_O (*g_s_*, 91.12 and 97.93%), transpiration rate (*E*, 66.57 and 85.66%), and instantaneous water use efficiency (WUEins, 23.97 and 78.08%), but increased leaf temperature (LT, by 12.9 and 17.88%) and intrinsic water use efficiency (WUEint, 189.31 and 50.97%) (Figure 2a–f). Contrariwise, impressively improved *P_n_* (by 447.91 and 1964.28%), *g_s_* (262.79 and 1094.64%), *E* (151.89 and 258.76%), WUEint (50.39 and 74.79%), and WUEins (119.29 and 476.781%), and decreased LT (16.01 and 28.23%) were found in ‘S1 + Eth’ and ‘S2 + Eth’ plants, respectively, relative to those values found in the corresponding ‘S1’ and ‘S2’ plants (Figure 2a–f). Additionally, as compared with the ‘Control’ plants, ‘Eth’ plants displayed enhancements in *P_n_* and *E* by 22.98 and 36.05%, respectively, but a decline in LT by 10.70% (Figure 2a,c,d).

### 2.4. Ethanol Safeguards Photosynthetic Pigments in Salt-Stressed Soybean Plants

**Figure 3:**
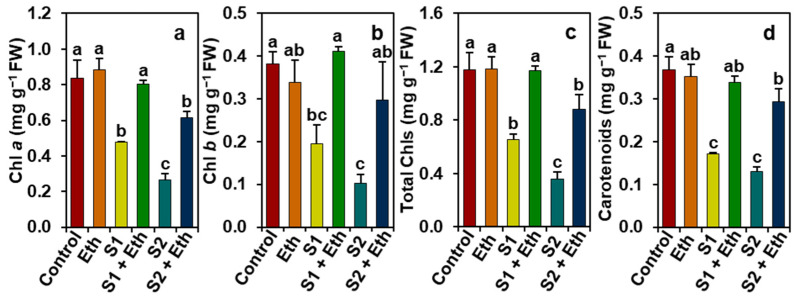
Effects of foliar-sprayed ethanol on the levels of (**a**) Chl *a*, (**b**) Chl *b*, (**c**) total Chls, and (**d**) carotenoids in leaves of soybean plants exposed to a gradient of salinity for 7 days. The statistically significant differences (*p* < 0.05) among various treatments are shown by different letters following the least significant difference (LSD) test. Chl, Chlorophyll; FW, fresh weight; Eth, 20 mM ethanol; S1, 8 dS m^−1^; S1 + Eth, 8 dS m^−1^ + 20 mM ethanol; S2, 16 dS m^−1^; S2 + Eth, 16 dS m^−1^ + 20 mM ethanol.

‘S1’ and ‘S2’ plants, respectively, exhibited notable reductions in the levels of Chl *a* (by 42.58 and 68.10%), Chl *b* (48.53 and 72.82%), total Chls (44.34 and 69.54%), and carotenoids (53.16 and 64.40%), when compared with those of the ‘Control’ plants (Figure 3a–d). By contrast, ‘S1 + Eth’ and ‘S2 + Eth’ plants, respectively, showed remarkable increases in the levels of Chl *a* (by 67.41 and 131.36%), Chl *b* (111.26 and 185.85%), total Chls (78.95 and 146.15%), and carotenoids (97.10 and 123.92%), corresponding to ‘S1’ and ‘S2’ plants (Figure 3a–d).

### 2.5. Ethanol Reduces Oxidative Damage in Soybean Plants under Salt Stress

**Figure 4:**
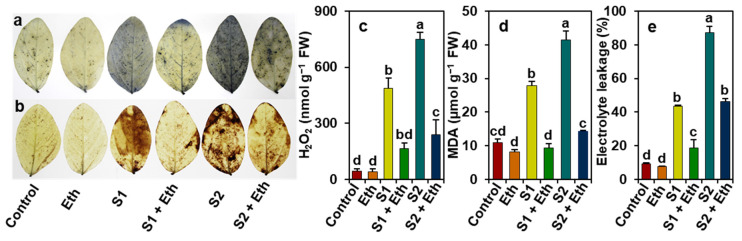
Effects of foliar-sprayed ethanol on reactive oxygen species accumulations in leaves of soybean plants exposed to a gradient of salinity for 7 days. (**a**) Superoxide (O_2_^•^^−^) and (**b**) hydrogen peroxide (H_2_O_2_) accumulations were stained with NBT and DAB solutions, respectively. Levels of (**c**) H_2_O_2_, (**d**) malondialdehyde (MDA), and (**e**) electrolyte leakage (EL) in the leaves of soybean plants. The statistically significant differences (*p* < 0.05) among various treatments are shown by different letters following the least significant difference (LSD) test. FW, fresh weight; Eth, 20 mM ethanol; S1, 8 dS m^−1^; S1 + Eth, 8 dS m^−1^ + 20 mM ethanol; S2, 16 dS m^−1^; S2 + Eth, 16 dS m^−1^ + 20 mM ethanol. NBT; nitroblue tetrazolium; DAB; 3,3’-diaminobenzidine.

Relative to the ‘Control’ plant leaves, staining of the leaves of ‘S1’ and ‘S2’ plants with nitro blue tetrazolium (NBT) for superoxide (O_2_^•−^) and 3,3’-diaminobenzidine (DAB) for hydrogen peroxide (H_2_O_2_) resulted in the development of more deep blue spots and dark brown spots, respectively (Figure 4a,b). By comparison, ‘S1 + Eth’ and ‘S2 + Eth’ plant leaves showed a notable reduction in the accumulation of O_2_^•−^ and H_2_O_2_, when contrasted with the corresponding ‘S1’ and ‘S2’ plant leaves (Figure 4a,b). More specifically, the leaves of ‘S1’ and ‘S2’ plants exhibited significant enhancements in the levels of H_2_O_2_ by 1016.50 and 1621.93%, malondialdehyde (MDA) by 154.92 and 277.87%, and electrolyte leakage (EL) by 361.44 and 823.76%, respectively, as compared with the level recorded in the ‘Control’ plant leaves (Figure 4c–e). In contrast, reduced contents of H_2_O_2_ (by 65.95 and 67.82%), MDA (66.67 and 65.40%), and EL (57.26 and 46.93%) were found in ‘S1 + Eth’ and ‘S2 + Eth’ plant leaves, respectively, when compared with the corresponding values in ‘S1’ and ‘S2’ plant leaves (Figure 4c–e).

### 2.6. Ethanol Improves Antioxidant Defense Responses in Salt-Stressed Soybean Plants

**Figure 5:**
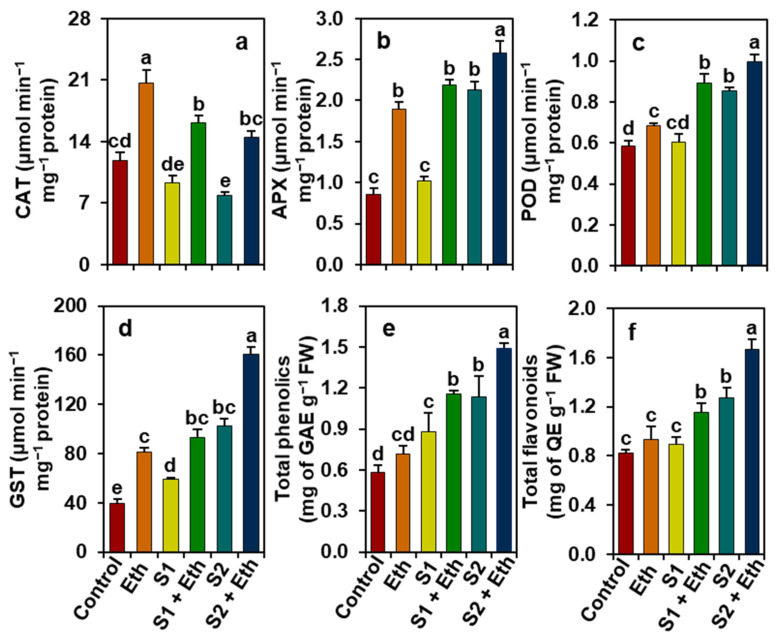
Effects of foliar-sprayed ethanol on (**a**) catalase (CAT), (**b**) ascorbate peroxidase (APX), (**c**) peroxidase (POD), and (**d**) glutathione *S*-transferase (GST) activities, and the levels of (**e**) total phenolics and (**f**) total flavonoids in leaves of soybean plants exposed to a gradient of salinity for 7 days. The statistically significant differences (*p* < 0.05) among various treatments are shown by different letters following the least significant difference (LSD) test. FW, fresh weight; Eth, 20 mM ethanol; S1, 8 dS m^−1^; S1 + Eth, 8 dS m^−1^ + 20 mM ethanol; S2, 16 dS m^−1^; S2 + Eth, 16 dS m^−1^ + 20 mM ethanol; GAE, gallic acid equivalent; QE, quercetin equivalent.

Relative to the ‘Control’ plants, ‘S1’ and ‘S2’ plants showed enhancements in the activities of APX (by 148.46% in ‘S2’), peroxidase (POD, 46.27% in ‘S2’), and GST (47.71 and 157.84%, respectively) (Figure 5b–d). However, the activities of CAT decreased by 21.45 and 33.20% in ‘S1’ and ‘S2’ plants, respectively, compared with that found in the ‘Control’ plants (Figure 5a). In contrast, notably improved activities of CAT (by 73.33 and 83.55%), APX (114.66 and 20.73%), POD (140.38 and 16.83%), and GST (58.34 and 56.37%) were observed in ‘S1 + Eth’ and ‘S2 + Eth’ plants, respectively, in contrast to those observed in ‘S1’ and ‘S2’ plants (Figure 5a–d). In addition, ‘Eth’ plants also showed significant enhancements in the activities of CAT, APX, POD, and GST by 73.89, 121.14, 17.41, and 104.25%, respectively, relative to the ‘Control’ plants (Figure 5a–d).

On the other hand, ‘S1’ and ‘S2’ plants showed noteworthy augmentations in the levels of total phenolics (by 50.73 and 94.63%, respectively) and total flavonoids (55.22% in ‘S2’), when compared with the ‘Control’ plants (Figure 5e,f). By comparison, further enhancements in the content of total phenolics (by 31.36 and 30.87%) and total flavonoids (29.97 and 30.86%) were found in ‘S1 + Eth’ and ‘S2 + Eth’ plants, respectively, unlike in those observed in ‘S1’ and ‘S2’ plants (Figure 5e,f).

### 2.7. Ethanol Enhances the Levels of Osmoprotectants in Salt-Stressed Soybean Plants

In relation to the ‘Control’ plants, ‘S1’ and ‘S2’ plants showed lower levels of leaf relative water content (RWC, by 38.15 and 59.33%, respectively) and water-soluble protein (27.09% in ‘S2’), but higher levels of Pro (by 3039.72 and 1335.32%, respectively), total free amino acids (116.25% in ’S2’), and total soluble sugars (252.53 and 299.07%, respectively) (Table 1). In contrast, ‘S1 + Eth’ and ‘S2 + Eth’ plants, respectively, displayed significantly higher levels of leaf RWC (by 33.03 and 90.78%), Pro (50.49 and 91.44%), and total free amino acids (58.96 and 33.10%), but lower levels of water-soluble protein (by 53.92 and 35.02%) and total soluble sugars (50.69 and 44.72%) than the corresponding salt-stressed plants only (Table 1). Additionally, the levels of water-soluble proteins in ‘Eth’ plants decreased by 42.85% when compared with the ‘Control’ plants (Table 1).

### 2.8. Ethanol Maintains Mineral Balance in Salt-Stressed Soybean Plants

**Figure 6:**
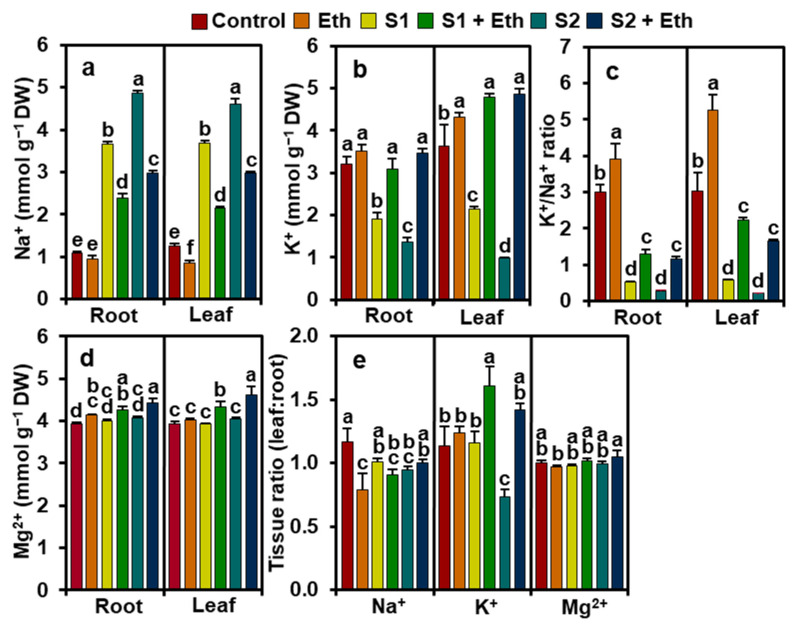
The content of different nutrients and tissue mineral ratios (leaf: root) among different parts of soybean plants exposed to a gradient of salinity for 7 days. Levels of (**a**) Na^+^, (**b**) K^+^, (**c**) K^+^/Na^+^ ratio, and (**d**) Mg^2+^, and tissue ratios of (**e**) Na^+^, K^+^, and Mg^2+^ for leaf/root of soybean plants. Values (means ± SEs) of each treatment were attained from six biological replications (*n =* 6). The statistically significant differences (*p* < 0.05) among various treatments are shown by different letters following the least significant difference (LSD) test. DW, dry weight; Eth, 20 mM ethanol; S1, 8 dS m^−1^; S1 + Eth, 8 dS m^−1^ + 20 mM ethanol; S2, 16 dS m^−1^; S2 + Eth, 16 dS m^−1^ + 20 mM ethanol.

Relative to ‘Control’ plants’ roots, ‘S1’ and ‘S2’ plants’ roots, respectively, showed a significant increase in Na^+^ content (by 239.61 and 350.57%) and a decrease in potassium ion (K^+^) content (40.36 and 57.18%). Consequently, notable reductions in root K^+^/Na^+^ ratios (by 82.56 and 90.56%, respectively) were observed in ‘S1’ and ‘S2’ plants, relative to the ‘Control’ plants (Figure 6a–c). By comparison, ‘S1 + Eth’ and ‘S2 + Eth’ plants’ roots, respectively, exhibited a reduced level of Na^+^ (by 34.78 and 38.87%) and an augmented level of K^+^ (62.61 and 151.85%), resulting in a higher K^+^/Na^+^ ratio (by 150.49 and 312.58%), when compared with the corresponding values recorded in the roots of ‘S1’ and ‘S2’ plants (Figure 6a–c). However, the levels of magnesium ions (Mg^2+^) remained comparable between ‘S1’ and ‘S2’, and ‘Control’ plants (Figure 6d). However, ‘S1 + Eth’ and ‘S2 + Eth’ plants’ roots displayed an increase in Mg^2+^ levels by 6.42 and 8.77%, respectively, in comparison to the corresponding ‘S1’ and ‘S2’ plants (Figure 6d). In ‘Eth’ plants, the root Mg^2+^ content significantly increased by 5.66% compared to that in the ‘Control’ plants; however, Na^+^ and K^+^ levels remained comparable between ‘Eth’ and ‘Control’ plants (Figure 6a,b,d). Interestingly, in ‘Eth’ plants, the K^+^/Na^+^ ratio in the roots increased by 31.02% compared to that in the ‘Control’ plants (Figure 6c).

The leaves of ‘S1’ and ‘S2’ plants, respectively, exhibited a significant upsurge in Na^+^ content (by 196.11 and 270.43%) and a decline in K^+^ content (by 40.72 and 73.19%), resulting in a lower K^+^/Na^+^ ratio (by 80.75 and 93.01%), as compared with the observed values in the ‘Control’ plants (Figure 6a–c). In contrast, ethanol supplementation attenuated Na^+^ content (by 41.89 and 35.63%) and enhanced K^+^ content (122.39 and 399.69%) in the leaves of ‘S1 + Eth’ and ‘S2 + Eth’ plants, respectively, resulting in an increase in the K^+^/Na^+^ ratio by 283.01 and 673.66%, respectively, when compared with the ‘Control’ plants (Figure 6a–c). In addition, ‘S1 + Eth’ and ‘S2 + Eth’ plants also showed an enhanced Mg^2+^ content in the leaves by 10.31 and 14.23%, respectively, in relation to the ‘Control’ plants (Figure 6d). Likewise, in comparison with the ‘Control’ plants, ‘Eth’ plants showed a reduced accumulation of Na^+^ by 31.83%, an increased accumulation of K^+^ by 18.87%, and consequently, a higher K^+^/Na^+^ ratio by 73.10% in the leaves (Figure 6a–c).

Furthermore, compared with those of the ‘Control’ plants, ion ratios within different plant organs showed that ‘S1’ and ‘S2’ plants displayed a reduced leaf/root ratio of Na^+^ content (by 18.87% in ‘S2’) and K^+^ content (by 35.55 in ‘S2’), while in the case of Mg^2+^, the reduction was not significant for the same treatments (Figure 6e). Interestingly, in ‘S1 + Eth’ and ‘S2 + Eth’ plants, a remarkably reduced leaf/root ratio of Na^+^ content (by 10.18% in ‘S1’) and an increased leaf/root ratio of K^+^ content (38.29 and 93.58%, respectively) was observed, when compared with those of corresponding values found in ‘S1’ and ‘S2’ plants (Figure 6e). Additionally, in comparison with the ‘Control’ plants, ‘Eth’ plants also displayed a lower leaf/root ratio of Na^+^ content (by 32.27%) (Figure 6e).

### 2.9. Clustering Heatmap-Based Data Visualization under Different Treatments and Treatment-Parameter Association by Principal Component Analysis

**Figure 7:**
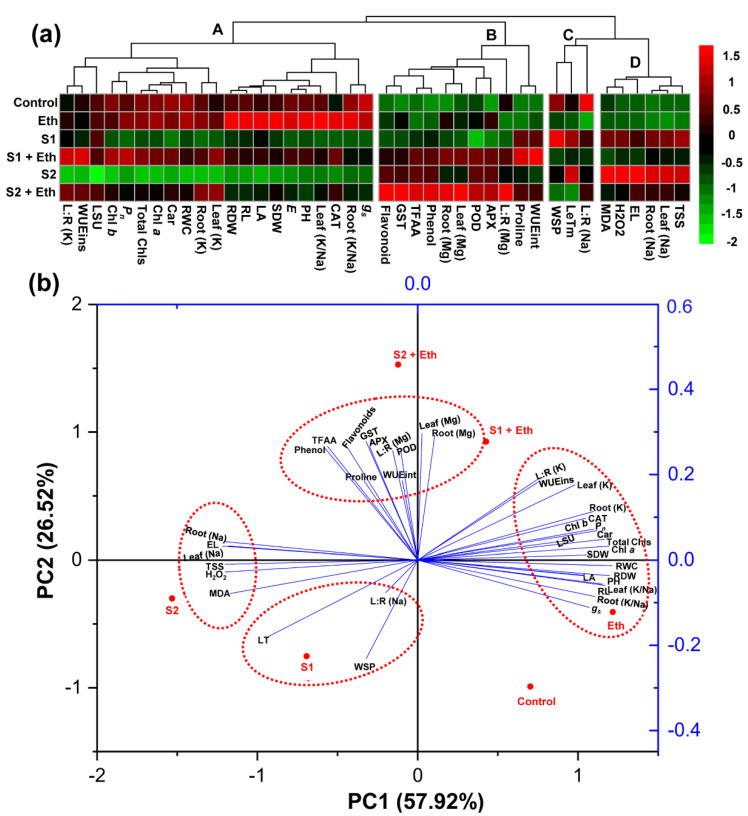
(**a**) Clustering heatmap visualizing different parameters under different treatments at a glance. Normalized mean values of different parameters were used to prepare the heatmap. The parameters were grouped into four distinct clusters. (**b**) Principal component analysis (PCA) represents the relationship among the different treatments and parameters. The biplot was created with the first two components (PC1 and PC2) that collectively explains 84.44% of the variability among the datasets. The vector lines of the biplot display positive or negative associations of different morpho-physiological and biochemical parameters with different treatments. The angle value between the parameter and treatment specifies the intensity of association between treatment and subsequent parameter, where a small angle indicates a weak association, and a large angle indicates a strong association. The parameters included RDW (root dry weight), RL (root length), LA (total leaf area per trifoliate), Leaf (K/Na) (leaf K^+^ and Na^+^ ratio), SDW (shoot dry weight), *E* (transpiration rate), PH (plant height), CAT (catalase), Total Chls (total chlorophylls), Chl *a* (chlorophyll *a*), Chl *b* (chlorophyll *b*), Car (carotenoids), RWC (relative water content), *P_n_* (photosynthetic rate), *g_s_* (conductance to H_2_O), Leaf (K) (leaf K^+^ content), LSU (leaf succulence), WUEins (instantaneous water-use efficiency), Root (Mg) (root Mg^2+^ content), Leaf (Mg) (leaf Mg^2+^ content), L:R (K) (leaf and root ratio of K^+^ content), L:R (Mg) (leaf and root ratio of Mg^2+^ content), Root (Na) (root Na^+^ content), APX (ascorbate peroxidase), TFAA (total free amino acids), Phenol (total phenolics), POD (peroxidase), Flavonoids (total flavonoids), GST (glutathione *S*-transferase), Pro (proline), WUEint (intrinsic water-use efficiency), WSP (water-soluble protein), LT (leaf temperature), Root (K/Na) (root K^+^ and Na^+^ ratio), Root (K) (root K^+^ content), MDA (malondialdehyde), H_2_O_2_ (hydrogen peroxide), EL (electrolyte leakage), L:R (Na) (leaf and root ratio of Na^+^ content), Leaf (Na) (leaf Na^+^ content), and TSS (total soluble sugars). Eth, ethanol; S1, 8 dS m^−1^; S1 + Eth, 8 dS m^−1^ + 20 mM ethanol; S2, 16 dS m^−1^; S2 + Eth, 16 dS m^−1^ + 20 mM ethanol.

A heatmap was generated to visualize the performance of different parameters under different treatment conditions using color intensity, and the parameters were further grouped into four different clusters using the hierarchical clustering method (Figure 7a). When compared with the ‘Control’ plants, the parameters of cluster-A revealed a declining trend in ‘S1’ and ‘S2’ plants. Interestingly, ‘S1 + Eth,’ ‘S2 + Eth,’ and ‘Eth’ plants displayed a reverse trend than those of ‘S1’ and ‘S2’ plants for cluster-A parameters, with the exception of Chl *b*, carotenoids, and WUEins in ‘Eth’ plants (Figure 7a). In comparison to the ‘Control’ plants, most of the variables in cluster-B exhibited an upward trend under both levels of salt stress. However, there were further escalations in the levels of these parameters in ‘S1 + Eth,’ ‘S2 + Eth,’ and ‘Eth’ plants, with exceptions in the cases of Mg^2+^ for leaf/root and total free amino acids in ‘Eth’ plants (Figure 7a). When compared with the respective ‘Control’ plants, parameters of cluster-C exhibited a changeable trend under saline conditions; nevertheless, all of the features of this cluster declined upon ethanol treatment (Figure 7a). In comparison with the ‘control’ plants, parameters of cluster-D displayed an increasing tendency under stress conditions; however, foliar ethanol application reverses the trend in both salt-stressed and non-stressed plants (Figure 7a). Subsequently, to find out the association between different treatments and variables, principal component analysis (PCA) was carried out (Figure 7b). The PC1 (57.92%) and PC2 (26.52%) accounted for the majority of the variability and collectively explained 84.44% of the variability. Notably, parameters of cluster-A and cluster-B were found to have a close association with ‘S1 + Eth’ and ‘S2 + Eth’ treatments, while cluster-B and -C variables were found to be closely related to ‘S1’ and ‘S2’ treatments (Figure 7a,b). Nonetheless, ‘Eth’ plants showed a close relationship with cluster-A and -B variables instead of cluster-C and -D variables (Figure 7a,b).

## 3. Discussion

Soil salinity has emerged as a severe environmental problem that has a variety of detrimental effects on plant growth and development [6,21]. In this study, we found that soybean plants subjected to salt stress displayed a distortion of morphological features, including the wilting and yellowing of leaves, as well as reductions in root length, shoot height, shoot DW and root DW, leaf area per trifoliate, and leaf succulence, when compared with control conditions (Figure 1a–j). These results were corroborated with previous findings in other salt-stressed legume crops, such as lentil (*Lens culinaris*) and mung bean (*Vigna radiata*) [6,22]. Conversely, the foliar application of ethanol to salt-stressed plants resulted in decreased canopy wilting and yellowing, as well as restored growth rate and biomass production, thereby playing a decisive role in alleviating salt-mediated deleterious effects in soybean plants (Figure 1a–j). Our findings were further supported by PCA, which revealed that salt-stressed soybean plants supplemented with ethanol had a less negative interaction with growth features than ethanol-devoid stressed plants (Figure 7b). The positive regulatory role of ethanol in enhancing plant growth performance has also been reported by Rowe et al. [23], Yavarpanah et al. [24], and Nguyen et al. [20].

The impairment of phenotype and reduction in growth and biomass might be a consequence of abnormal photosynthesis (Figure 2a), which could be mediated by the salt-induced destruction of photosynthetic pigments as a result of an enhancement of ROS production [25,26]. Our results showed that salt-stressed soybean plants had substantially lower levels of photosynthetic pigments (Figure 3a–d), whereas the application of exogenous ethanol to salt-exposed plants retained photosynthetic pigment levels (e.g., Chls and carotenoids) and net photosynthetic rates, compared with salt-stressed plants only (Figure 2a and Figure 3a–d). These findings suggest that ethanol may have a positive role in the prevention and/or delaying of the destruction of photosynthetic pigments (Figure 3a–d), resulting in an enhancement of photosynthesis capacity of soybean plants under salinity (Figure 2a), which was supported by the findings of Nguyen et al. [20]. In addition, the improved photosynthetic rate in ethanol-sprayed salt-stressed soybean plants might be associated with a greater leaf area per trifoliate (Figure 1i), which ensures the maximum light interception capacity that ultimately boosts the photosynthetic rate in plants [27,28,29]. It is worth noting that ethanol enabled salt-stressed soybean plants to retain greater photosynthesis by boosting their WUE under the physiological drought circumstances, as evidenced by higher levels of WUEint and WUEins, leading to more biomass gain than salt-stressed plants only. Interestingly, higher transpiration rates in ethanol-supplemented salt-stressed plants contributed to the maintenance of leaf cooling, as evidenced by their lower LT than the plants stressed with salt only (Figure 3c,d). Our PCA analysis also provided compelling evidence that the beneficial effects of external ethanol in salt-stressed soybean plants were positively correlated with improved photosynthesis and WUE (Figure 7b).

Our findings also revealed that salt stress led to an increased accumulation of ROS products, including O_2_^•−^ and H_2_O_2_ (Figure 4a,b, respectively). These results together with high levels of MDA and EL indicated a greater degree of membrane damage in salt-stressed soybean plants (Figure 4a–e). The addition of exogenous ethanol, on the other hand, helped minimize the burden of ROS-mediated oxidative damage and provided protection against cell membrane damage, as evidenced by diminished levels of ROS, MDA, and EL in salt-stressed soybean leaf tissues (Figure 4a–e). In line with our findings, PCA analysis also demonstrated a negative interaction between the treatments of ethanol-sprayed soybean plants and the levels of H_2_O_2_, MDA, and EL; however, a positive association was observed in water-sprayed salt-treated plants (Figure 7b).

Plants have evolved a robust antioxidant defense mechanism that includes both enzymatic and nonenzymatic antioxidants to combat ROS-induced oxidative damage under salt stress [2,30]. In the current study, we determined the activities of several key enzymes and the levels of nonenzymatic antioxidants such as total phenolics and flavonoids to identify the effective roles of ethanol in oxidative stress mitigation. Our findings showed that water-sprayed salt-exposed plants enhanced the activity of APX, GST, and POD; however, the activity of CAT was observed to be decreased (Figure 5a–d). The ethanol-supplemented salt-stressed soybean plants, on the other hand, further increased the activities of APX, POD, GST, and CAT, compared with salt-stressed plants alone (Figure 5a–d). It is plausible that the elevated activities of CAT, APX, and POD greatly contributed to the reduction in oxidative damage by detoxifying H_2_O_2_ in the leaves of ethanol-added salt-stressed soybean plants. Furthermore, GST is an important enzymatic player for activating glutathione-dependent peroxide-detoxification system, which ensures greater protection against lipid hydroperoxides, reactive aldehydes and ketones, and organic peroxides produced from the effects of high salinity [30]. In previous findings, transcriptome analyses of ethanol-treated salt-exposed *Arabidopsis* plants revealed the upregulation of several genes involved in the regulation of ROS homeostasis under salinity, including *AtAPX1*, *AtAPX2*, *AtGSTU4*, and *AtGSTU19* [20]. Our findings further demonstrated that ethanol addition boosted the levels of nonenzymatic antioxidants, including total phenolics and flavonoids, in salt-stressed soybean plants (Figure 5e,f), which might have played a crucial role in safeguarding the cell membrane from oxidative damage by scavenging toxic ROS during salinity stress [6,31,32,33]. Our results were supported by the PCA analysis, which demonstrated a substantial positive correlation between the treatments of ethanol-supplemented salt-stressed plants and the activities and/or levels of enzymatic and nonenzymatic antioxidants (Figure 7b).

Plants produce a wide array of osmoprotectants to support osmotic balance under salinity stress [34]. The results of the current study disclosed that excessive salt stress resulted in a significant accumulation of free amino acids, Pro, and total soluble sugars in soybean plants (Table 1). Intriguingly, ethanol treatment of salt-stressed soybean plants further increased the levels of Pro and free amino acids, but reduced the levels of soluble sugars when contrasted with that in salt-stressed plants alone (Table 1). Likewise, the PCA biplot revealed a robust and positive relationship between foliar ethanol application on salt-stressed soybean plants and the levels of the osmoprotectants, Pro and total free amino acids, whereas total soluble sugar exhibited a negative relationship (Figure 7b). The augmented level of Pro in ethanol-supplied salt-stressed soybean plants might help in retaining the water status of the plant, as reflected by higher levels of leaf succulence and RWC (Figure 1j, Table 1). In addition, Pro might provide protection to photosynthetic machineries, cell membranes, and protein functions by scavenging ROS [35,36,37]. An enhanced accumulation of free amino acids, on the other hand, aided plants in maintaining optimum protein synthesis by supplying an adequate supply of amino acids [27,38,39]. In accordance with our findings, a positive correlation between improved salt tolerance and the levels of free amino acids and Pro has also been reported in faba bean (*Vicia faba*) and *V*. *radiata* [6,35].

Importantly, the poor growth performance of soybean plants under salt stress might be a consequence of an imbalanced nutrient distribution, as manifested by the greater accumulation of toxic Na^+^ and a notable decline in beneficial K^+^ levels (Figure 1 and Figure 6a,b). A salt-induced nutrient imbalance was further evidenced from PCA biplot analysis, which showed the strong and positive associations between salt stress treatments and Na^+^ accumulation in different tissues of soybean plants (Figure 7b). Intriguingly, adding ethanol to salt-treated plants notably enhanced the levels of K^+^ and Mg^2+^ while abating Na^+^ levels in both leaves and roots (Figure 6a,b,d). In addition, a lower leaf/root ratio of Na^+^ but a higher leaf/root ratio of K^+^ in ethanol-supplemented salt-stressed soybean plants also indicated a preferential nutrient allocation for maintaining better growth under saline conditions (Figure 6e). Therefore, ethanol-mediated ion homeostasis provided an indirect but strong indication that ethanol played a pivotal role in effective Na^+^ sequestration into the vacuoles, which was further substantiated by increased leaf succulence (Figure 1j). Plants with succulent features have the ability to dilute absorbed salts in their succulent leaves and, thus, protect the metabolically active cellular compartments by restricting the excessive buildup of Na^+^ ions in them [27]. Improved K^+^ levels in leaves in ethanol-added salt-stressed soybean plants might assist in cell enlargement and optimal metabolic function, and preserve the structural integrity of proteins under ambient salt stress [40,41,42]. Moreover, increased Mg^2+^ levels in leaves may aid in protecting the chloroplast ultrastructure, translocating photoassimilates, and synthesizing chlorophylls, which has essential roles in the maintenance of optimum photosynthesis under saline conditions (Figure 6d) [43]. Thus, our results showed that the application of exogenous ethanol might efficiently ameliorate salinity-induced ion toxicity in soybean plants through the reduction in Na^+^ accumulation and preferential nutrient allocation within different plant parts, which, in turn, promotes the overall growth performance of soybean plants.

## 4. Materials and Methods

### 4.1. Plant species, Growth Environments, and Stress Treatments

A high-yielding (1.8–2.1 tons ha^−1^) soybean (BARI Soybean-6) variety was chosen to assess the roles of ethanol in the mitigation of salt stress. Healthy seeds were surface-sterilized using sodium hypochlorite solution (5%, *v*/*v*) containing Tween-20 solution (0.2%, *v*/*v*) for 20 min followed by washing three times with distilled water (dH_2_O). Next, the sterilized seeds were immersed in dH_2_O at room temperature in the dark for 8 h for imbibition. The seeds were then covered with a wet cloth for 48 h to allow the radicle to emerge. The well-emerged radicles were then planted in a 2.5 L plastic pot (17 cm height, 18 cm diameter) containing 2.5 kg of soil (eight radicles pot^−1^). Soils were prepared by mixing them with cow dung and sand in a weight-basis ratio of 2:1:0.5. Furadan, a well-known pesticide, was added to the soil (3.0 g kg^–1^ of soil) to prevent soil-borne diseases. On the 10^th^ day after sowing, 200 mL of diluted urea (4.0 g L^−1^ of water) was applied to each pot to ensure a sufficient supply of nitrogen fertilizer. Twelve-day-old seedlings at the vegetative V1 stage (completely formed first trifoliate) were divided into two groups (each group contained 3 pots). Both groups of pots were irrigated each day with tap water (control) and 8 dS m^−1^ (S1) and 16 dS m^−1^ (S2) of saline water (200 mL pot^−1^) for seven days (7 times in total). During 12:15 p.m. to 13:00 p.m., one group of pots was simultaneously foliar-sprayed (20 mL to each pot) with 20 mM of ethanol (Eth), while the pots from the remaining group were sprayed with tap water only (20 mL to each pot). Therefore, the present study consisted of six treatments, including (i) water-sprayed control (Control), (ii) 20 mM ethanol-sprayed control (Eth), (iii) water-sprayed 8 dS m^−1^ salt stress (S1), (iv) S1 + Eth, (v) water-sprayed 16 dS m^−1^ salt stress (S2), and (vi) S2 + Eth. Tween-20 (0.2%, *v*/*v*) was used as a surfactant to ensure the maximal adherence of ethanol to the leaves. Nineteen-day-old seedlings were harvested to evaluate the performance of soybean under the above-mentioned conditions. The first trifoliate leaves were collected to determine various physiological and biochemical attributes. The experiment was repeated thrice to ensure that the results were accurate.

### 4.2. Determination of Growth Parameters, RWC, and EL

Following the method of Rahman et al.’s [27], three plants were randomly selected from each treatment to appraise the morphological features, including the height of shoot and DW of the shoots and roots. Roots from both control and salt-stressed plants were carefully detached from the soil, followed by washing according to the procedure described in Rahman et al. [6]. Afterward, primary root length was determined using a measuring scale. The EL of detached soybean leaves was determined following the method of Kim et al. [44]. Leaf RWC was assessed as described by Mostofa et al. [45] with a slight modification. The leaves of the first trifoliate were excised, and the fresh weight (FW) was immediately recorded. Excised leaves were hydrated to full turgid by immersing them in deionized H_2_O for 4 h. The leaves were then gently pressed with tissue to remove adhered water, and the full turgid weight (TW) was taken. Oven-drying of the leaf samples was carried out at 80 °C for 72 h followed by determination of DW. The percentage of RWC was calculated according to the equation: RWC (%) = [(FW − DW)/(TW − DW)] × 100.

### 4.3. Measurement of Leaf Succulenceand Leaf Area per Trifoliate Leaf

The leaf succulence of freshly harvested trifoliate leaves was measured using the protocols specified by Rahman et al. [6]. Total leaf area per trifoliate was determined using Carleton and Foote’s [46] technique.

### 4.4. Quantification of Ion Contents

With the help of an atomic absorption spectrophotometer (PinAAcle 900H, Perkin Elmer, Waltham, MA, USA), the methods of Rahman et al. [14] was adopted to quantify the contents of Na^+^, K^+^, and Mg^2+^ in oven-dried samples of roots and leaves.

### 4.5. Gas Exchange Parameters

Between 10:30 a.m. and 12:30 p.m., the portable infrared gas analyzer system (LI-6400XT, LI-COR Inc., Lincoln, NE, USA) was used to measure the *P_n_*, *E*, *g_s_*, and LT in the fully developed first trifoliate leaf (count from the plant’s base). In addition, soybean leaves’ WUEint and WUEins were determined using the formulae described by Rahman et al. [14].

### 4.6. Determination of the Content of Photosynthetic Pigments and Pro

The amounts of Chl *a*, Chl *b*, and total Chls, as well as the levels of carotenoids in freshly harvested soybean leaves, were spectrophotometrically estimated using the techniques described by Arnon [47], and Lichtenthaler and Wellbura [48], respectively. In addition, Pro content in freshly collected leaves was determined according to the procedure outlined by Bates et al. [49].

### 4.7. Estimation of the Contents of Phenolics and Flavonoids

The levels of total phenolics and total flavonoids in soybean leaf tissues were determined following the procedures reported by Ainsworth and Gillespie [50], and Zhishen et al. [51], respectively, with slight modifications. Briefly, soybean leaf samples (0.1 g) were homogenized in 1.5 mL of methanol (100%) and centrifuged for 20 min at 4 °C at 11,500× *g.* The collected supernatant was utilized following two different methodologies to quantify total phenolics and flavonoid contents. To determine total phenolics, 0.15 mL of 10% Folin-Ciocalteu’s reagent was mixed with 0.3 mL of supernatant and kept at room temperature for 15 min. After that, 0.6 mL of 700 mM Na_2_CO_3_ was added to the above mixture and kept at room temperature for 120 min. Finally, the absorbance was recorded at 765 nm using a UV-VIS spectrophotometer (GENESYS 10S, Thermo Scientific, San Jose, CA, USA). For the measurement of flavonoids, 0.15 mL of 5% sodium nitrite, 0.22 mL of 10% AlCl_3_·6H_2_O, and 1.12 mL of 1.0 M NaOH were added to 0.3 mL of supernatant. The mixture was then shaken using a vortex mixer (XH-D Vortex Mixer, Shanghai Leewen Scientific Instrument Co. Ltd., Shanghai, China). Finally, the absorbance was measured at 510 nm using the above-mentioned spectrophotometer. Gallic acid and quercetin were used as standards for the determination of total phenolics and total flavonoids, respectively.

### 4.8. Histochemical Detection of O_2_^•^^−^ and H_2_O_2_, and Quantification of the Levels of H_2_O_2_ and MDA

After 9 days of stress treatment, the histochemical detection of O_2_^•−^ and H_2_O_2_ in soybean leaves was performed according to the methods of Mostofa and Fujita [52] with a slight modification. To stain O_2_^•−^ and H_2_O_2_, fully grown first trifoliate soybean leaves were soaked in 0.05% (*w*/*v*) NBT and DAB solutions, respectively. The leaves immersed in NBT and DAB were incubated for 24 h under dark and light conditions, respectively. After incubation, the green color of the leaves was decolorized in boiling ethanol. The developed blue and brown spots indicated the presence of O_2_^•−^ and H_2_O_2_, respectively. The photographs were taken by putting the stained soybean leaves on a whiteboard. The contents of H_2_O_2_ and lipid peroxidation product MDA in the first trifoliate leaves were quantified spectrophotometrically according to the methods reported by Yu et al. [53] and Kim et al. [44], respectively.

### 4.9. Preparation of Enzyme Supernatants and Determination of Enzyme Activities

The thorough procedures outlined by Rahman et al. [6] were followed for the preparation of enzyme extracts, and the estimation of the activities of CAT (EC 1.11.1.6), APX (EC: 1.11.1.11), POD (EC: 1.11.1.7), and GST (EC: 2.5.1.18).

### 4.10. Quantification of Water-Soluble Proteins, Free Amino Acids, and Soluble Sugars

The measurement of water-soluble protein contents in the enzyme extracts collected from the leaves of soybean plants were carried out following the spectrophotometric method of Bradford [54]. Total free amino acid and total soluble sugar contents were determined following the comprehensive procedures reported by Lee and Takahashi [55], and Somogyi [56], respectively.

### 4.11. Statistical Analysis

The obtained data were analyzed using one-way analysis of variance (ANOVA) with the employment of Statistix 10. The statistically significant differences (*p* < 0.05) among various treatments were shown by different letters following the least significant difference (LSD) test, using Statistix 10 software. Three biological replications (n = 3) were used to obtain the values (means ± SEs) of each treatment, and they are presented in the Figure and Tables. Principle component analysis (PCA) was conducted using OriginPro 2021 software. A clustering heatmap was created with the normalized mean values of different parameters using R Studio 1.4.1717.

## 5. Conclusions

From our findings, we can conclude that ethanol can effectively ameliorate salt-induced growth retardation and biomass loss by modulating multiple physiological and biochemical processes. More specifically, ethanol application aided in (i) improving leaf succulence; (ii) attenuating uptake, transport, and accumulations of toxic Na^+^; (iii) shielding photosynthetic pigments degradation to improve photosynthetic performance; (iv) reducing oxidative stress and cellular damage by restricting the accumulations of excessive ROS; (v) augmenting CAT, APX, POD, and GST activities along with improving total phenolics and flavonoids levels; and (vi) enhancing compatible solutes accumulation in soybean under salt stress conditions. It is likely that application of exogenous ethanol might be a sustainable and cost-effective solution for reducing salinity-induced adverse effects on soybean production to support sustainable agriculture in saline-affected areas. Nevertheless, a further in-depth field investigation using a range of crop species, and different salinity regimes and modes of ethanol applications would be required to determine the advantageous role of ethanol in the proper management of salinity problems. As ethanol may quickly convert to other active metabolites, a comprehensive metabolite profiling might aid in determining the true contributor to ethanol-mediated salt tolerance in soybeans. Importantly, it would be interesting to assess if ethanol supplementation positively affects seed biochemical constituents and nutritional values in soybean, which might help us better address malnutrition issues in underdeveloped countries.

## Figures and Tables

**Table 1:** Effects of exogenous ethanol in modulation of the levels of water content, free amino acids, proline, water-soluble proteins, and soluble sugars in the leaves of soybean plants exposed to a gradient of salinity for 7 days.

Treatment	Leaf Relative Water Content (%)	Total Free Amino Acids (µg g^−^^1^ FW)	Proline (μmol g^−^^1^ FW)	Water-Soluble Proteins (mg g^−1^ FW)	Total Soluble Sugars (mg g^−1^ FW)
Control	86.41 ± 3.59 ^ab^	45.54 ± 1.00 ^c^	0.63 ± 0.04 ^e^	7.06 ± 0.33 ^b^	9.93 ± 0.11 ^c^
Eth	88.92 ± 0.88 ^a^	43.95 ± 0.21 ^c^	0.74 ± 0.05 ^e^	4.04 ± 0.10 ^d^	8.32 ± 0.65 ^c^
S1	53.44 ± 4.96 ^d^	62.04 ± 0.52 ^c^	20.00 ± 0.87 ^b^	8.58 ± 0.09 ^a^	34.99 ± 2.64 ^a^
S1 + Eth	71.10 ± 1.75 ^bc^	98.60 ± 4.24 ^b^	30.10 ± 0.30 ^a^	3.95 ± 0.07 ^d^	17.26 ± 1.25 ^b^
S2	35.14 ± 9.24 ^e^	98.48 ± 17.91 ^b^	9.15 ± 0.81 ^d^	5.15 ± 0.08 ^c^	39.61 ± 3.17 ^a^
S2 + Eth	67.04 ± 1.26 ^cd^	131.08 ± 1.29 ^a^	17.50 ± 1.07 ^c^	3.35 ± 0.12 ^e^	21.90 ± 1.99 ^b^

Different alphabetical letters within the column indicate statistically significant differences among the treatments by a least significant difference test (*p* < 0.05). FW, fresh weight; Eth, 20 mM ethanol; S1, 8 dS m^−1^; S1 + Eth, 8 dS m^−1^ + 20 mM ethanol; S2, 16 dS m^−1^; S2 + Eth, 16 dS m^−1^ + 20 mM ethanol.

## Data Availability

The data presented in this study are available on request from the corresponding author. The data are not publicly available due to privacy.
